# Investigation of carbonyl amidation and *O*-methylation during biosynthesis of the pharmacophore pyridyl of antitumor piericidins

**DOI:** 10.1016/j.synbio.2022.05.001

**Published:** 2022-05-10

**Authors:** Wanlu Li, Wenyu Zhang, Yijia Cheng, Yaoyao Shen, Jianzhao Qi, Hou-Wen Lin, Yongjun Zhou

**Affiliations:** a Research Center for Marine Drugs, State Key Laboratory of Oncogenes and Related Genes, Department of Pharmacy, Ren Ji Hospital, School of Medicine, Shanghai Jiao Tong University, Shanghai, 200127, China; b Shaanxi Key Laboratory of Natural Products & Chemical Biology, College of Chemistry & Pharmacy, Northwest A&F University, Yangling, 712100, Shaanxi, China

**Keywords:** Polyketide, Pyridine, Polyketide synthase (PKS), Natural product biosynthesis

## Abstract

Piericidins are a large family of bacterial *α*-pyridone antibiotics with antitumor activities such as their anti-renal carcinoma activity exhibited recently in nude mice. The backbones of piericidins are derived from *β*, *δ*-diketo carboxylic acids, which are offloaded from a modular polyketide synthase (PKS) and putatively undergo a carbonyl amidation before *α*-pyridone ring formation. The tailoring modifications to the *α*-pyridone structure mainly include the verified hydroxylation and *O*-methylation of the C-4′ position and an unidentified C-5′ *O*-methylation. Here, we describe a piericidin producer, terrestrial *Streptomyces conglobatus*, which contains a piericidin biosynthetic gene cluster in two different loci. Deletion of the amidotransferase gene *pie*D resulted in the accumulation of two fatty acids that should be degraded from the nascent carboxylic acid released by the PKS, supporting the carbonyl amidation function of PieD during *α*-pyridone ring formation. Deletion of the *O*-methyltransferase gene *pie*B1 led to the production of three piericidin analogues lacking C-5′ *O*-methylation, therefore confirming that PieB1 specifically catalyses the tailoring modification. Moreover, bioactivity analysis of the mutant-derived products provided clues regarding the structure-function relationship for antitumor activity. The work addresses two previously unidentified steps involved in pyridyl pharmacophore formation during piericidin biosynthesis, facilitating the rational bioengineering of the biosynthetic pathway towards valuable antitumor agents.

## 1 Introduction

**Fig. 1:**
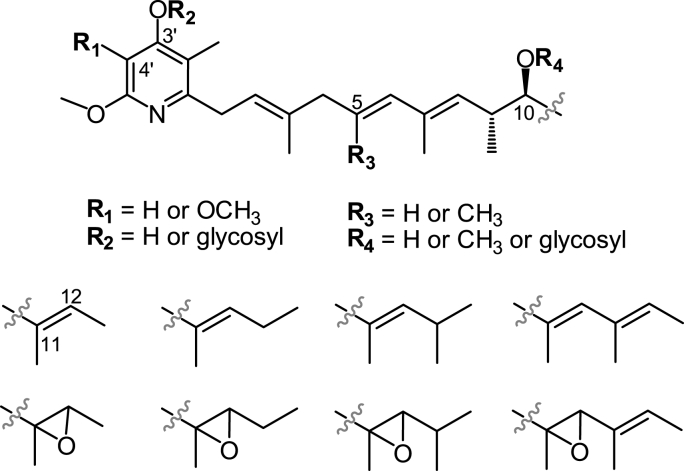
Representative structural variations of natural piericidins.

Piericidins are a large family of bacterial natural products featuring with a pyridone core linked to a linear polyene chain, representing some of the most commonly encountered compounds in natural product studies of *Actinomycetes* species collected from soil, insect symbiosis, and marine samples [[1], [2], [3], [4], [5]]. The structural diversity of natural piericidins is mainly derived from the variable polyene side chain and substitutions in the pyridyl core [6] (Fig. 1). Due to their bioactive potential against microorganisms, insects, and tumor cells, eighty-nine biosynthetic and eleven chemosynthetic piericidin analogues have been discovered thus far [7]. The activity of piericidins was highlighted again recently by piericidin A1, the firstly discovered member of this family. The compound was verified to induce apoptosis in renal carcinoma cells by reducing the ROS level caused by the upregulated peroxiredoxin 1 (PRDX1) and also demonstrated potent antitumor efficacy in renal carcinoma xenograft mice [8,9].

**Fig. 2:**
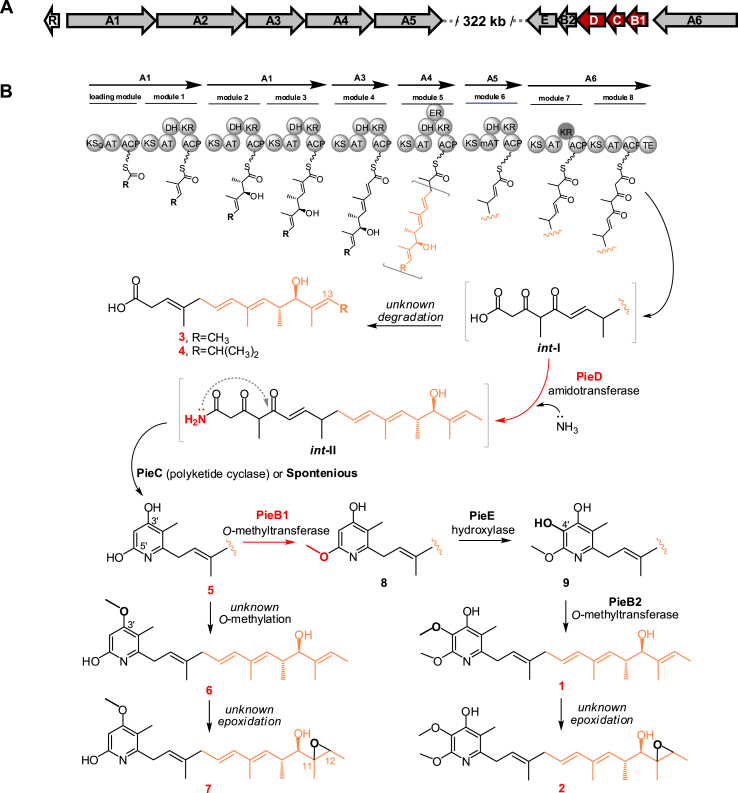
Genetic organization of *pie*BGC from *S. conglobatus* (A) and the proposed biosynthetic pathway of compounds **1**–**7** (B). The compounds isolated in this study are indicated by red numbers.

The piericidin biosynthetic gene cluster (*pie*BGC) was successively investigated in two *Streptomyces* species [2,10,11], and the biosynthesis of piericidin backbone was elucidated as follows: 1) the modular polyketide synthase (PKS) machinery of PieA1-A6 releases a polyene chain with a *β*, *δ*-diketo carboxylic acid terminal group after nine rounds of 2-carbon assembly; 2) the nascent polyene chain is amidated at the terminal carboxylic acid by the ATP-dependent amidotransferase PieD; 3) the amidated product undergoes cyclization spontaneously or driven by the polyketide cyclase PieC to form an *α*-pyridone ring (Fig. 2B). The pyridyl structure of piericidin was then verified to receive the tailoring modifications of C-4′ hydroxylation by the FAD-dependent monooxygenase PieE and C-4′ *O*-methylation by the *O*-methyltransferase PieB2 [10,11], whereas the C-5′ *O*-methylation putatively conducted by the *O*-methyltransferase PieB1 remains unverified (Fig. 2B). Moreover, there are additional unconventional modifications such as the obscure epoxidation of the polyene side chain and *O*-glycosylation of either the *α*-pyridone core or polyene side chain [12] (Fig. 1). Despite extensive efforts, at least two points remain to be identified in the piericidins biosynthetic pathway; the putative amidation function of PieD and the pyridyl C-5′ *O*-methylation thought to be conducted by PieB1 due to the lack of the accumulation of new intermediates after individual inactivation of the *pie*D and *pie*B1 genes [2,10].

We recently discovered that terrestrial *Streptomyces conglobatus* can produce piericidins, as this species contains a *pie*BGC with the genes found in two different loci of the genome (Fig. 2A). The construction of gene deletion mutants in this new piericidin producer and structural elucidation of the intermediates accumulated by mutant strains allowed us to address the carbonyl amidation step that is essential for the formation of the *α*-pyridone ring pharmacophore and the tailoring *O*-methylation modification at the C-5′ of pyridyl core. Moreover, we investigated a structure-activity relationship by analyzing the antitumor activities of the five compounds isolated from mutant strains. This work further clarifies the piericidin biosynthetic pathway to facilitate rational bioengineering towards accumulating valuable antitumor agents.

## 2 Materials and methods

### 2.1 DNA manipulation and chemicals

The oligonucleotides used in this work were ordered from Shanghai Generay Biotech Co., Ltd. Restriction endonucleases and T4 DNA ligase were purchased from New England Biolabs. Chemicals were purchased from Sigma–Aldrich. The Plasmid DNA Extraction Kit was purchased from Shanghai Generay Biotech. DNA fragments were assembled by using the U–Clone master mix kit (Evomic Science, Sunnyvale, CA, USA). Genomic DNA was prepared by using 10% Chelex 100 resin (Bio-Rad) solution. PCR amplifications were carried out by using Phusion High-Fidelity PCR Master Mix (New England Biolabs) for DNA cloning and 2 × Flash PCR MasterMix (CWBIO) for colony screening.

### 2.2 Strains, media, and culture conditions

Strain RJ8 is a mutant of terrestrial *S. conglobatus* [14] and was used as the starting strain/wild type strain in this work. *E. coli* DH10B was used as a host for plasmid construction. *E. coli* ET12567 containing the helper plasmid pUZ8002 was used as a transitional host for introducing the *E. coli*−*Streptomyces* shuttle plasmid into RJ8. TSBY medium (3% tryptone soy broth, 0.5% yeast extract, 10% sucrose, 0.1% antifoam) was used to culture mycelium of RJ8 or derived strains. SFM agar medium (2% soya flour, 2% D-mannitol, 2% agar) was used for manipulation of conjugation and mutant screening from the RJ8 strain. SGC medium consisting of 3% soybean flour, 5% glucose, 0.5% CaCO_3_, and 0.1% (v/v) antifoam was used as the medium for secondary metabolite production. *Streptomyces* fermentation was conducted in a 250 mL conical flask fitted with a metal spring, with 50 mL of SGC medium inoculated with 1% (v/v) of 3 d TSBY culture, and then incubated at 30 °C, 220 rpm for 5 d. *E. coli* strains were grown in Luria-Bertani (LB) broth (1% tryptone, 0.5% yeast extract, 0.5% NaCl) or an LB agar (1.5% agar) plate at 37 °C supplemented with the corresponding antibiotics.

### 2.3 Analytical methods

For routine HPLC–MS analysis, 7 mL of five-day fermentation broth was extracted with an equal volume of ethyl acetate. The extract was dried via vacuum evaporation and redissolved into 500 *μ*L of methanol. Then, 20 *μ*L of the final sample was injected into the HPLC–MS for analysis.

HPLC–MS analysis was conducted on an HPLC coupled with a Waters Acquity QDa detector. The HPLC was fitted with a Waters Xbridge C18 column (250 × 4.6 mm, 5 *μ*m). Samples were eluted with the mobile phases of acetonitrile and H_2_O (0.1% formic acid, v/v) at a flow rate of 0.7 mL/min with a gradient elution of 30–95% acetonitrile over 30 min. The mass spectrometer was run in positive ionization mode, scanning from *m*/*z* 200 to 1250.

HRMS spectra were acquired with a UPLC Waters XeVO G2-XS Q TOF mass spectrometer in positive ionization mode, scanning from *m*/*z* 100 to 1200. MS/MS analysis was conducted with a collision energy ramp of 30–40 eV, scan time (sec) of 0.200, and interscan time (sec) of 0.014. The UPLC was fitted with a Waters Acquity UPLC BEH C18 column (2.1 mm × 50 mm, 1.7 *μ*m). A mobile phase of acetonitrile and H_2_O (0.1% formic acid, v/v) was used for isocratic sample elution with 60% acetonitrile over 5 min at a flow rate of 0.4 mL/min.

NMR spectra were recorded on a Bruker AV-600 MHz NMR spectrometer in CDCl_3_ or DMSO-*d*_*6*_ with TMS as an internal standard.

### 2.4 Deletion of *pie*B1CD to generate the mutant RJ10

To generate the platform mutant RJ10, the whole ORFs of the *pie*B1CD genes were selected for deletion from the RJ8 strain, which lost the ability to produce the dominant secondary metabolites of conglobatin and neoantimycins after inactivation of *cong*E and *nat*E from *S. conglobatus* [14,15]. To construct a homologous recombination cassette, primers Lpie-S and Lpie-A and primers Rpie-S and Rpie-A (Table S1) were used to amplify a 1578 bp left arm and a 1630 bp right arm, respectively. By using the U-Clone master mix kit, the two PCR fragments were assembled with the *E. coli*−*Streptomyces* shuttle plasmid pRJ2 linearized with *Xba*I and *Eco*RI [14,15]. The resulting plasmid pRJ32 was transformed into the RJ8 strain via conjugation from *E. coli* ET12567 with the helper plasmid pUZ8002. The target mutant was screened by using colony PCR with the primers Tpie-SS and Tpie-AA (Table S1) from the hygromycin-sensitive colonies prepared after two rounds of propagation on SFM plates without antibiotics added. The expected PCR products were 443 bp for the target mutation and 3560 bp for the wild type strain RJ8. The PCR product of the mutant was sequenced for final confirmation.

### 2.5 Complementation of RJ10 with different compositions of the *pieB1*, *pieC*, and *pieD* genes

To identify the functions of the *pie*B1, *pie*C, and *pie*D genes in RJ10, the ORFs of *pie*B1CD, *pie*CD, *pie*B1C, and *pie*D were constructed under the constitutive promoter KasO*p [16], respectively, based on the integrative vector pRJ5 [17]. In detail, the KasO*p promoter containing fragments (167–170 bp) was amplified from pRJ255 [17] (see primers in Table S1). The gene transcription frames consisting of 3172 bp (*pie*B1CD), 2441 bp (*pie*CD), and 1906 bp (*pie*D) were amplified from the genomic DNA of RJ8 (see primers in Table S1). By using U-Clone master mix kit, the KasO*p promoter fragment and the corresponding ORF fragment were assembled with the linearized plasmid pRJ5 (NsiI, EcoRV) to yield the plasmids pRJ261 for *pie*B1CD, pRJ262 for *pie*CD, and pRJ263 for *pie*D. Moreover, the 1447 bp KasO*p-*pie*B1C cassette was amplified from pRJ261 and introduced into the NsiI and EcoRV sites of pRJ5 to produce the plasmid pRJ267 (see primers in Table S1).

The resultant plasmids pRJ261, pRJ262, pRJ263, and pRJ267 were then introduced into RJ10 via conjugation. The exconjugants were confirmed via the phenotype of hygromycin resistance on SFM plates and subsequent colony PCR (see primers in Table S1).

### 2.6 Purification of compounds 1–7

Compound production was conducted by using RJ8 for **1** and **2**, RJ10 for **3** and **4**, and *pie*CD/RJ10 for **5**–**7**. The 12 L of fermentation broth for each strain was extracted three times with an equal volume of ethyl acetate after adjusting the pH to 6.0 with formic acid. The combined organic phases were concentrated under vacuum, and subjected to vacuum liquid chromatography with silica gel (200–300 mesh) for fractionation by using stepwise elution with dichloromethane-methanol (from 50:1 to 0:1, v/v). Guided by HPLC–MS analysis, the fractions containing the expected compounds were combined and delivered for further separation through an ODS chromatography column (YMC-Pack Pro C18 RS, 20 × 250 mm, 5 *μ*m) by using medium-pressure preparative liquid chromatography at a flow rate of 15 mL/min with a gradient of 30–95% acetonitrile/H_2_O (0.1% formic acid) for 4 h, detection at a wavelength of 254 nm. Finally, the target fractions were purified by using HPLC fitted with a semipreparative column (Waters Xbridge C18, 10 × 250 mm, 5 *μ*m) with 80% methanol/H_2_O (0.1% formic acid) as the eluent and a flow rate of 3 mL/min.

### 2.7 Protein expression and purification of *PieB2*

The *pie*B2 gene was amplified from the genomic DNA of RJ8 (see primers in Table S1). The PCR fragments of *pie*B2 were introduced into the *Nde*I and *Xho*I sites of pET29a by using the U-Clone master mix kit, yielding the plasmid pRJ284. The resultant plasmid was transformed into *E. coli* BL21 (DE3) for protein expression. The overnight culture (1 mL) of the target transformant was inoculated into 500 mL of LB medium supplemented with kanamycin (50 *μ*g/mL) (37 °C, 220 rpm). Protein expression was induced by adding isopropyl-*β*-d-thiogalactopyranoside (IPTG) (0.2 mM) when cell turbidity reached to an OD_600_ of 0.6–0.8, followed by 15 h of cultivation at 22 °C. Cells were harvested by centrifugation (11,325×*g*, 5 min) and then resuspended in buffer A (50 mM Tris HCl, 300 mM NaCl, pH 7.2) for sonication. The supernatant of the cell lysate (34,925×*g*, 25 min of centrifugation) was passed through a 0.22 *μ*m filter before being loaded onto a His-Bind affinity column (1 mL bed volume). Target proteins were eluted via stepwise increases in the concentration of imidazole (10–500 mM). Concentration and buffer exchange of the target protein was performed by using Amicon Ultra-4 concentrators (Millipore, 10 kDa cut-off). Protein-containing fractions were analyzed by Bis-Tris Gel SDS–PAGE (4–12%). The protein concentration was measured with a NanoDrop 1000 spectrophotometer.

### 2.8 *PieB2* enzyme assays

The enzymatic assay was carried out in a 100 *μ*L reaction system with 100 *μ*M compound **5**, 10 *μ*M PieB2, and 2 mM *S*-adenosylmethionine in 50 mM Tris-HCl buffer (pH 8.0) and incubated at 28 °C for 5 h PieB2 preboiled at 100 °C for 10 min was used as a negative control. The reactions were stopped by adding 400 *μ*L of acetonitrile, and the solutions were centrifuged at maximum speed for 5 min before taking 20 *μ*L of the supernatant for HPLC–MS analysis.

### 2.9 Bioactivity assays of compounds 1–7

For the antimicrobial activity assay, the minimum inhibition concentration (MIC) values of **1**–**7** were evaluated using the 96-well microtiter plate method [18]. The tested compounds were dissolved in DMSO at a concentration of 3200 *μ*g/mL. The compounds used as positive controls were vancomycin for MRSA (CICC10201) and *B. mycoides*, rifampicin for *M. smegmatis* mc2155, and amphotericin B for *C. albicans*. DMSO was used as a negative control in the assay.

A cytotoxicity assay was conducted by using HL-60, A549, SMMC-7721, MDA-MB-231, and SW480 human cancer cells. The cells were incubated in RPMI-1640 medium for 12–24 h at 37 °C before the tested compounds were added and then incubated for an additional 48 h. For the first evaluation, each compound was used at a concentration of 40 *μ*M, and for determining the IC_50_ values (half-inhibitory concentrations), a gradient of compound concentrations was used. Cell viability was determined by adding MTS solution and incubating the cells at 37 °C for 4 h. The amount of the formazan crystal products dissolved in DMSO was detected with a 570 nm in spectrophotometer.

## 3 Results and discussion

### 3.1 Discovery of piericidins and bioinformatics analysis of *pieBGC from S. conglobatus*

Terrestrial *S. conglobatus* was previously shown to produce the antitumor macrodiolide conglobatin and cyclic depsipeptide neoantimycins [14,19]. During a screening of neoantimycin analogues from the culture of *S. conglobatus*, we found two components that were structurally identical to piericidins A1 (**1**) and C1 (**2**) according to comparative analysis of the ^13^C and ^1^H NMR data (Tables S2 and S3). Interestingly, genome mining revealed that the expected *pie*BGC should be composed of the two clusters of genes located at a distance of 322 kb in the genome of *S. conglobatus* (GenBank OM674914) (Fig. 2A). The left part of the *pie*BGC contains five PKS genes, *pie*A1-A5, which are responsible for the loading starter unit and six two-carbon elongations. The right portion includes the *pie*A6 gene encoding the last two modules of the piericidin PKS, and the *pie*B1, C, D, B2, and E genes putatively involved in the *α*-pyridine ring formation and tailoring modifications (Fig. 2A). Except for the different physical locations, the newly found *pie*BGC displays the same gene organization and a high level of protein homology compared to the reported *pie*BGC from *Streptomyces piomogeues* and *Streptomyces* sp. SCSIO 03032 [2,10] (Table 1), therefore suggesting a correlation to the production of **1** and **2**.

**Table 1:** Deduced functions of ORFs in *pie*BGC from *S. conglobatus*.

Gene	Size (aa)	Proposed function	Protein Homolog (Accession No.)	Identity/Coverage
**R**	266	Transcriptional regulator, SARP family	PieR (AHE80990.1)	71/96
**A1**	2743	type I PKS (AT-ACP, KS-AT-DH-KR-ACP)	PieA1 (AEZ54374.1)	56/98
**A2**	3468	type I PKS (KS-AT-KR-ACP, KS-AT-DH-KR-ACP)	PieA2 (AEZ54375.1)	62/100
**A3**	1896	type I PKS (KS-AT-DH-KR-ACP)	PieA3 (AHE80993.1)	57/100
**A4**	2201	type I PKS (KS-AT-DH-ER-KR-ACP)	PieA4 (AEZ54377.1)	69/95
**A5**	1923	type I PKS (KS-AT-DH-KR-ACP)	PieA5 (AEZ54378.1)	65/100
**A6**	2982	type I PKS (KS-AT-KR-ACP, KS-AT-ACP-TE)	PieA6 (AEZ54379.1)	54/100
**B1**	230	*O*-methyltransferase	PieB1 (AEZ54380.1)	71/98
**C**	177	Polyketide cyclase	PieC (AEZ54381.1)	76/92
**D**	616	Amidotransferase	PieD (AEZ54382.1)	79/95
**B2**	264	*O*-methyltransferase	PieB2 (AEZ54383.1)	75/97
**E**	611	FAD dependent hydroxylase	PieE (AEZ54384.1)	75/95

### 3.2 Deletion of *pieB1CD* resulted in the accumulation of three new products

**Fig. 3:**
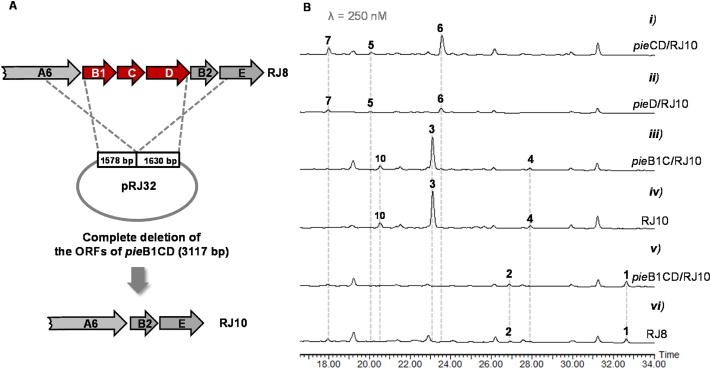
Deletion of *pie*B1CD ORFs via double-crossover homologous recombination (A), and HPLC–MS analysis of the metabolites of the resultant mutant and corresponding gene complementation strains (B). The compound numbers are used to label only the detected products from each set of HPLC–MS data based on extracting the target mass.

The putative ATP-dependent amidotransferase encoded by *pie*D in *pie*BGC was assumed to transfer a nitrogen from ammonia or glutamine to the terminal *β*, *δ*-diketo carboxylic acid group of the nascent C_18_ polyene chain offloaded from the PKS assembly line of PieA1*–*A6, and the amidated product could undergo *α*-pyridine ring cyclization either spontaneously or driven by a putative cyclase encoded by *pie*C [2] (Fig. 2B). In a previous experiment, the *pie*D gene was inactivated by gene replacement with an apramycin resistance cassette. However, the resultant Δ*pie*D strain did not accumulate new products such as linear polyene carboxylic acids, although the production of **1** was destroyed as expected [2]. To probe the function of PieD, we designed a mutant with the open reading frame (ORF) of the *pie*B1CD gene cassette deleted from strain RJ8 (Fig. 3A), which was created from *S. conglobatus* by removing the dominant products conglobatin and neoantimycins [14,20].

According to HPLC–MS analysis of the fermentation extract, the resultant Δ*pie*B1CD mutant strain (RJ10) lost the ability to produce **1** and **2** as expected. Surprisingly, three new products, **3**, **4**, and **10**, were accumulated in RJ10 with a considerable yield of **3** (Fig. 3B, iv). When *pie*B1CD cassette was delivered back to RJ10 via an integrative vector, the production of **1** and **2** was recovered to the same level as that of the parent strain RJ8, and the three new compounds disappeared in the fermentation culture (Fig. 3B, v and vi). When the *pie*B1 and *pie*C genes were introduced into RJ10, the resultant strain *pie*B1C/RJ10 (Δ*pie*D) gave the same metabolite profile as RJ10 (Fig. 3B, iii), therefore confirming that the accumulation of **3**, **4**, and **10** was directly correlated with the absent function of PieD.

### 3.3 Structural elucidation of 3 and 4 accumulated by the *ΔpieD* strain

To characterize the structures of the three new products accumulated by the Δ*pie*D strain, we performed a 24-L liquid fermentation to purify enough of each compound for spectral analysis. Finally, 27 mg of compound **3** and 9 mg of compound **4** were obtained as yellow oils; however, the instability of compound **10** during the isolation procedure prevented its quantitative preparation.

**Fig. 4:**
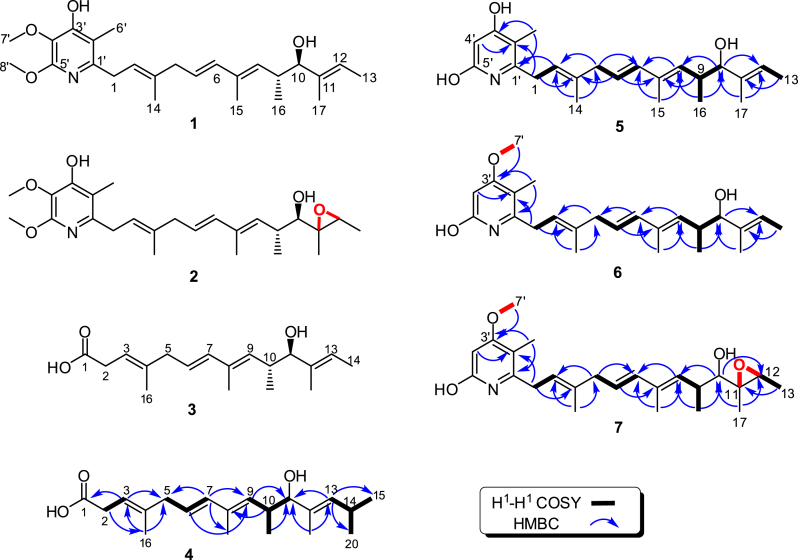
The structures of **1**–**7** and the ^1^H–^1^H COSY and HMBC correlations of **4**–**7**.

According to analysis of the ^1^H and ^13^C NMR data, **3** should be structurally identical to a reported fatty acid polyketide isolated from marine-derived *S. youssoufiensis* OUC6819 [20] (Fig. 4, Table S4). The molecular formula of **4** was determined to be C_20_H_32_O_3_ based on its HRESIMS ion at *m/z* 343.2427 [M+H]^+^ (calc. for HC_20_H_32_O_3_^+^, 343.2424, 0.93 ppm). Detailed interpretation of the NMR data revealed that **4** is a derivative of **3** with two additional methyl groups at C-14 (*δ*_C_ 26.2). This was confirmed by the HMBC correlations from H-13 (*δ*_H_ 5.08) to C-15 (*δ*_C_ 22.8) and C-20 (*δ*_C_ 23.0) in **4** (Fig. 4, Table S5). Moreover, the configurations of four double bonds in **4** were confirmed as *E* by NOESY correlations between H-11/H-13, H-7/H-9, H-10/H-17, H-6/H-17, H-2/H-16, and H-14/H-19. The relative configurations of C-10 and C-11 were proposed to be trans based on the coupling constant values of 7.1 Hz (Fig. 4, Table S5). The molecular formula of **10** was determined to be C_25_H_36_O_5_ based on its HRMS ion at *m/z* 417.2630 [M+H]^+^ (calc. for HC_25_H_36_O_5_^+^, 417.2636, −1.43 ppm).

We speculate that **3** and **4** could be oxidatively degraded from the unstable *β*,*δ*-diketo carboxylic acids offloaded from the piericidin PKS, in which the starter units of acetate and isobutyrate were incorporated to yield **3** and **4**, respectively (Fig. 2B), similar to the piericidin analogues derived from diverse starter precursors in nature (Fig. 1) [6]. Interestingly, the structure of **3** is identical to a compound discovered from the piericidin producer *S. youssoufiensis* OUC6819 [13,20], implying that **3** could be naturally produced with the inefficient activity of PieD. We hypothesized that compound **10** could be an *α*-pyrone product (Fig. S1) that was formed spontaneously from the instable *β*,*δ*-diketo carboxylic acid nascently offloaded from the PKS, in terms of the evidence that the *α*-pyrone ring was produced efficiently *in vitro* by the last module of the piericidin PKS with an inactivated C-terminal TE [2]. In conclusion, the accumulation of **3**, **4** and **10** in the Δ*pie*D mutant provides indications for the previously speculated PieD-catalyzed carbonyl amidation of the carboxylic acid nascently released by the piericidin PKS before *α*-pyridone ring cyclization.

### **3.4** Deletion of *pieB1* or *pieB1C* resulted in the accumulation of 5–7

In a previous study, based on gene disruption and enzymatic assay, the *O*-methyltransferase gene *pie*B2 was verified to conduct *O*-methylation at the C-4′ hydroxyl group generated by FAD-dependent monooxygenase PieE in the piericidin pyridyl core [10] (Fig. 2B). However, due to the lack of new intermediates detected in the Δ*pie*B1 mutant, it remains to be confirmed whether another *O*-methyltransferase gene, *pie*B1, performs *O*-methylation of the C-5′ hydroxyl group [10]. To probe the function of *pie*B1 in our piericidin producer, we created a Δ*pie*B1 mutant through complementation of RJ10 with the *pie*C and *pie*D genes based on an integrative vector. Interestingly, the Δ*pie*B1 strain (*pie*CD/RJ10) produced three new products, **5***–***7**, with considerable yield for **6** according to HPLC–MS analysis of the fermentation extract (Fig. 3B, i). We speculate that the failed accumulation of **5***–***7** in the reported Δ*pie*B1 strain may be attributed to an interruption in the transcript cassette *pie*B1CDB2E, in which the *pie*B1 gene was destroyed by a fragment consisting of the apramycin resistance gene *aac*(3)IV and the conjugation gene *ori*T [10].

We also confirmed the function of PieC by creating a Δ*pie*B1C mutant, which was generated by complementation of RJ10 with the *pie*D gene. According to HPLC–MS analysis, the production of **5***–***7** was remarkably reduced in Δ*pie*B1C compared to the production levels in Δ*pie*B1 strain (Fig. 3B, i and ii), therefore supporting the proposal that PieC acted as a polyketide cyclase to increase the rate of *α*-pyridine ring cyclization during piericidin biosynthesis [2]. Moreover, compounds **3** and **4** were not detected in *pie*CD/RJ10 or pieD/RJ10 strain according to LC-MS analysis, indicating that PieD could convert int-I into int-II quite specifically and efficiently so that even trace amount of int-I was not remained to be degraded into **3** or **4** (Fig. 2B).

### **3.5** Structural elucidation of 5–7 to identify the function of *PieB1*

To prepare adequate samples of **5***–***7** for structure characterization, the liquid fermentation broth of the Δ*pie*B1 mutant strain (*pie*CD/RJ10) was scaled up to 12-L. Subsequent chromatographic separations yielded yellow oil compounds including 3 mg of **5**, 13 mg of **6**, and 30 mg of **7**. Based on HRESIMS and NMR analyses, the planar structures of **5***–***7** were identified as new piericidin analogues without hydroxylation at C-4′ and *O*-methylation of the C-5′ hydroxyl group in the *α*-pyridone core of **1** or **2** (Fig. 4).

The molecular formula of **5** was determined to be C_23_H_33_NO_3_ according to the HRMS ion at *m*/*z* 394.2455 [M+Na]^+^ (calc. for NaC_23_H_33_NO_3_^+^, 394.2353, 0.76 ppm). Based on ^1^H and ^13^C NMR analysis (Table S6), **5** contains the same skeleton as the reported piericidin analogue Mer-A2026B [21,22], which possesses an additional methoxy group at the C-5′ hydroxyl compared to **5**. The structure of **5** was further confirmed by 2D NMR, in particular with the use of the HMBC spectrum (Fig. 4).

The molecular formula of **6** was determined to be C_24_H_35_NO_3_ according to the HRESIMS ion at *m*/*z* 408.2520 [M+Na]^+^ (calc. for NaC_24_H_35_NO_3_^+^, 408.2509, 2.7 ppm). Comparison of ^1^H and ^13^C NMR data revealed that **5** and **6** had the same skeleton (Table S6). The only difference between the two was that the C-3′ hydroxyl in **5** was substituted by a methoxy in **6**. This difference was validated by the HMBC correlations from H-7′ (*δ*_H_ 3.78) to C-3′ (*δ*_C_ 169.40) in **6** (Fig. 4, Table S6, Fig. S9E). The geometries of oleﬁns in **6** were deduced as 2*E*, 5*E*, 7*E*, and 11*E* based on the NOESY correlations of H-1 to H-14, H-8 to H-6, and H-10 to H-12, and the large ^1^H–^1^H coupling constant of 15.5 Hz between H-5 and H-6 (Table S6, Fig. S9F). A large coupling constant (9.0 Hz) suggested an anti-relationship between H-9 (*δ*_H_ 2.67) and H-10 (*δ*_H_ 3.62) in **6**, and the relative conﬁguration of 9R* and 10R* was supported by the NOESY correlations of H-8/H-10/H-16/H-17/H-9 (Fig. S9F).

The molecular formula of **7** was determined as C_24_H_35_NO_4_ according to the HRESIMS ion at *m/z* 424.2477 [M+Na]^+^ (calc. for NaC_24_H_35_NO_4_^+^, 424.2458, 4.4 ppm). The NMR data of **7** were highly similar to those of **6** (Table S6), with the only differences being that two oleﬁnic carbon signals of *δ*_C_ 137.77 for C-11 and 119.44 for C-12 in **6** were replaced by an oxymethine (*δ*_H_ 2.92 H-12, *δ*_C_ 58.49 C-12) and an oxygenated quaternary carbon (*δ*_C_ 62.62 C-11) in **7**, suggesting the presence of a C-11/C-12 epoxy ring in **7**, which was further supported by the HMBC correlations from H-13 (*δ*_H_ 1.28) to C-11 (*δ*_C_ 62.62), H-17 (*δ*_H_ 1.27) to C-12 (*δ*_C_ 58.49), and H-10 (*δ*_H_ 2.88) to C-11 (*δ*_C_ 62.62) and C-12 (*δ*_C_ 58.49) (Fig. 4, Fig. S10F).

Taken together, the nonmethylated C-5′ hydroxyl groups in **5***–***7** suggest that PieB1 was specific for C-5′ *O*-methylation, complementary to the previous results that PieE carries out for C-4′ hydroxylation and PieB2 conducts C-4′ *O*-methylation [10] (Fig. 2B). Conclusively, C-5′ *O*-methylation by PieB1 occurred before C-4′ hydroxylation by PieE, and last, PieB2 performed C-4′ *O*-methylation in the tailoring modifications of piericidin biosynthesis (Fig. 2B).

### 3.6 Investigation of the activity of *PieB2* for C-3′ *O*-methylation

The C-3′ *O*-methylation in **6** or **7** suggests an unidentified *O*-methylation step in the tailoring modifications of piericidin biosynthesis. Similarly, the C-3′ *O*-methylated version of **1** was recently discovered from a marine-derived *Streptomyces* (Peng et al., 2021). We first suspected that PieB2 possessed promiscuous activity for C-3′ *O*-methylation to convert **5** to **6**. To probe this activity, we obtained the soluble proteins of *C*-His_6_-fused PieB2 from *E. coli* BL21 (DE3) harboring the pET29a-derived expression plasmid pRJ284. The proteins were purified to near homogeneity via Ni-NTA chromatography (Fig. S2). However, no conversion of **5** to **6** was observed in the assay containing 100 *μ*M **5**, 10 *μ*M PieB2, and 2 mM *S*-adenosylmethionine (SAM) in Tris−HCl buffer (50 mM, pH 8.0) incubated at 28 °C for 5 h. Considering the widespread of *O*-methyltransferase in bacterial secondary metabolites biosynthesis [23], we suppose that an *O*-methyltransferase elsewhere could generate the C-3′ *O*-methylation of **6** and **7**, being similar to the cryptic occurrence of C-10 *O*-methylation in piericidins (Fig. 1). Moreover, the C-11/C-12 epoxy ring in **7** could be formed by a monooxygenase, e.g. epoxidase and P450 monooxygenase, since that the epoxidations of alkenes in natural products are conducted by various monooxygenases [24].

### 3.7 The bioactivities of 3–7

The antitumor activities of **3***–***7** were evaluated in five human cancer cell lines including leukemia HL-60, lung cancer A549, liver cancer SMMC-7721, breast cancer MDA-MB-231, and colon cancer SW480 cells. Except for the weak inhibitory activity against SMMC-7721 cells (IC_50_ 26.93 ± 2.48) detected for **6**, the cell inhibition rates of **3***–***7** at a concentration of 40 *μ*M were all less than 50%. Moreover, only weak antimicrobial activities (MICs, above 100 *μ*g/mL) of **3*–*7** were determined against pathogenic microorganisms such as methicillin-resistant *Staphylococcus aureus* (MRSA), *Bacillus mycoides*, *Mycobacterium smegmatis*, and *Candida albicans*. Of note, excellent antiproliferative activity (IC_50_, 80 nM) in the HL-60 cell line was detected with an analogue of **6**, piericidin N produced by mangrove sediment-derived *S. psammoticus* SCSIO NS126 [25], which contains a C-5′ methoxyl instead of the C-3′ methoxyl group in **6** (Fig. S3), implying that the position of *O*-methylation in the pyridyl core should play an important role in the antitumor activities of piericidins.

## 4 Conclusion

The work has addressed two key steps in the biosynthetic pathway of piericidins with antitumor activity, including carbonyl amidation before pharmacophore *α*-pyridone ring formation and the tailoring C-5′ *O*-methylation modification in the *α*-pyridone core, both of which were not identified in previous investigations because no new products were detected with the same mutations of other piericidin producers. Moreover, we elucidated a structure-activity relationship by analyzing the antitumor activities of the five compounds isolated from the mutant strains generated in this work. Clarification of the piericidin biosynthetic pathway would facilitate the creation of diverse piericidin analogues for antitumor agent screening and the targeted accumulation of valuable components via rational bioengineering.

## CRediT authorship contribution statement

**Wanlu Li:** Data curation, Formal analysis, Investigation, Software, Visualization, Writing – original draft. **Wenyu Zhang:** Data curation, Formal analysis, Investigation, Software, Visualization, Writing – original draft. **Yijia Cheng:** Formal analysis, Investigation. **Yaoyao Shen:** Supervision. **Jianzhao Qi:** Funding acquisition, Writing – review & editing. **Hou-Wen Lin:** Resources. **Yongjun Zhou:** Conceptualization, Funding acquisition, Methodology, Project administration, Resources, Supervision, Validation, Writing – original draft, Writing – review & editing.

## Declaration of competing interest

Authors declare that they have no conflict of interest.
