# Supplementary material for: Investigation of carbonyl amidation and O-methylation during biosynthesis of the pharmacophore pyridyl of antitumor piericidins
Source: Synth Syst Biotechnol. 2022 May 10;7(3):880–6. doi: 10.1016/j.synbio.2022.05.001 (PMC9112059; doi:10.1016/j.synbio.2022.05.001)
Supplement: Multimedia component 1 [file mmc1.docx]

**Supporting Information for**

**Investigation of the carbonyl amidation and *O*-methylation in the pharmacophore pyridyl formation of antitumor piericidins biosynthesis**

| **Table S1.** The oligonucleotides and the related plasmids in the work | | | |
| --- | --- | --- | --- |
| **Primer** | **Sequence** **(5’ -> 3’)** | **Description** | |
| The homologous recombination cassette in **pRJ32** for generation of **RJ10 mutant (∆*pie*B1CD)** | | | |
| Lpie-S | ATCCCCGGGGACCTGCAGGTCGACTCTCGGCACTGGTCCAGGAC  (the underline: 25 bp overlapping with the XbaI side of pRJ2) | 1578 bp left arm | |
| Lpie-A | TTAGGAAGGGAGGAGAAGGGGTTGTCGCCTTTCATGAGGGGTTGGAG |  |  |
| Rpie-S | TCATGAAAGGCGACAACCCCTTCTCCTCCCTTCCTAAGGAG | 1630 bp right arm | |
| Rpie-A | TATCACGAGGCCCTTTCGTCTTCAAGGAACACCCACTCGTTCCAC  (the underline: 26 bp overlapping with the EcoR I side of pRJ2) |  |  |
| To identify the target mutation in **RJ10** | | | |
| Tpie-SS | TTGCGACCTCCTCCAACCCCTC | 443 bp product | |
| Tpie-AA | TGTTCCAGGGTGTCGCAGCAGTAG |  |  |
| ***pie*B1CD** genes under *KasO**p promoter based on pRJ5 (NsiI, EcoRV) to generate **pRJ261** | | | |
| KasOp-S | TGTAAAACGACGGCCAGTGCCAAGCTTGGGCTG  (the underline:33 bp overlapping with the Nsil side of pRJ5) | 167 bp *KasO**p promoter amplified from pRJ255 | |
| BCD-KasO-A | TGTGGTCGATCGCCATAACTCCCCCAGTCCTG |  |  |
| BCD-KasO-S | TGCAGGACTGGGGGAGTTATGGCGATCGACCACAG | 3172 bp *pie*B1CD amplified from RJ8 | |
| pie-KasO-A | AAACAGCTATGACATGATTACGAATTCGATAGAAGGGTCAGGAGAGTTTGATG (the underline:30 bp overlapping with the EcoRV side of pRJ5) |  |  |
| \| ***pie*CD** genes under *KasO**p promoter based on pRJ5 (NsiI, EcoRV) to generate **pRJ262** \| \| --- \| | | | |
| KasOp-S | TGTAAAACGACGGCCAGTGCCAAGCTTGGGCTG  (the underline:33 bp overlapping with the Nsil side of pRJ5) | 168 bp *KasO**p promoter amplified from pRJ255 | |
| CD-KasO-A | TACCAGCGGTTGCCCACAACTCCCCCAGTCCTG |  |  |
| CD-KasO-S | TGCAGGACTGGGGGAGTTGTGGGCAACCGCTGGTAC | 2441 bp *pie*CD amplified from RJ8 | |
| pie-KasO-A | AAACAGCTATGACATGATTACGAATTCGATAGAAGGGTCAGGAGAGTTTGATG (the underline:30 bp overlapping with the EcoRV side of pRJ5) |  |  |
| ***pie*D** gene under *KasO**p promoter based on pRJ5 (NsiI, EcoRV) to generate **pRJ263** | | | |
| KasOp-S | TGTAAAACGACGGCCAGTGCCAAGCTTGGGCTG  (the underline:33 bp overlapping with the Nsil side of pRJ5) | 170 bp *KasO**p promoter amplified from pRJ255 | |
| D-KasO-A | ATCCGGCGATTCCACACATAACTCCCCCAGTCCTGCAC |  |  |
| D-KasO-S | TGCAGGACTGGGGGAGTTATGTGTGGAATCGCCGGATG | 1906 bp *pie*D amplified from RJ8 | |
| pie-KasO-A | AAACAGCTATGACATGATTACGAATTCGATAGAAGGGTCAGGAGAGTTTGATG (the underline:30 bp overlapping with the EcoRV side of pRJ5) |  |  |
| ***pie*B1C** genes under *KasO**p promoter based on pRJ5 (NsiI, EcoRV) to generate **pRJ267** | | | |
| KasOp-S | TGTAAAACGACGGCCAGTGCCAAGCTTGGGCTG  (the underline:33 bp overlapping with the Nsil side of pRJ5) | 1447 bp KasO*p -*pie*B1C amplified from pRJ261 | |
| BC-KasO-A | TATGACATGATTACGAATTCGATAGAAGGGCTCAGTGCCCCTTCCCCGTGGAGTTG |  |  |
| Identification of the presence of pRJ261, pRJ262 or pRJ263 in RJ10 | | | |
| T-kasO-S | TTGTAAAGTCGTGGCCAGGAG | 1790 bp for pRJ261; 1059 bp for pRJ262; 524 bp for pRJ263 | |
| T-pieD-A | TCCTTGGTGGGGTAGTAGTACAG |  |  |
| Identification of the presence of pRJ267 in RJ10 | | | |
| T-kasO-S | TTGTAAAGTCGTGGCCAGGAG | 1059 bp for pRJ267 | |
| T-pieC-A | TCGACGACGTAGTCCTCGATCA |  |  |
| **PieB2** protein expression **(C-terminal 6×His tag)** based on **pRJ284** | | | |
| P-B2-S | ACTTTAAGAAGGAGATATACATATGGCGGTTGACAACGACCTGTAC  (the underline:21 bp overlapping with the Ndel side of pET-29a) | | 857 bp PCR product amplified from RJ8 was cloned in pET-29a (Ndel, Xhol). |
| P-B2-A | TCAGTGGTGGTGGTGGTGGTGCTCAGTGGTGGTGGTGGTGGTGGCGGCCGGCATGGGTCTCCCCCTC  (the underline:22 bp overlapping with the Xhol side of pET-29a) | |  |

| **Table S2.** Comparison of the ^1^H and ^13^C data of **1** and the reported piericidin A1 | | | | |
| --- | --- | --- | --- | --- |
| **Position** | **1** (DMSO-*d*_6_) | | piericidin A1 **^a^** (CDCl_3_) | |
|  | *δ*_C_ (600 MHz) | *δ*_H_ (*J* in Hz) (150 MHz) | *δ*_C_ (100 MHz) | *δ*_H_ (*J* in Hz) (25 MHz) |
| 1 | 34.01 | 3.28, d | 34.5 | 3.30, d |
| 2 | 121.97 | 5.32, t | 122.4 | 5.30, t |
| 3 | 134.26 |  | 134.7 |  |
| 4 | 42.33 | 2.73, d | 43.2 | 2.73, d |
| 5 | 124.46 | 5.46, m | 126.7 | 5.45, m |
| 6 | 136.28 | 6.01, d | 135.9 | 6.00, m |
| 7 | 135.16 |  | 134.7 |  |
| 8 | 132.1 | 5.27, d | 133.2 | 5.12, d |
| 9 | 36.1 | 2.53, t | 37.0 | 2.66, m |
| 10 | 80.52 | 3.60, d | 82.9 | 3.53, d |
| 11 | 137.77 |  | 135.9 |  |
| 12 | 121.97 | 5.33, q | 123.3 | 5.35, q |
| 13 | 12.71 | 1.54, d | 13.1 | 1.57, d |
| 14 | 11.3 | 1.65, s | 10.7 | 1.68, s |
| 15 | 17.89 | 0.76, d | 17.5 | 0.76, d |
| 16 | 16.42 | 1.50, s | 16.6 | 1.54, s |
| 17 | 12.78 | 1.69, s | 13.1 | 1.70, s |
| 1’ | 149.69 |  | 150.8 |  |
| 2’ | 112.68 |  | 112.2 |  |
| 3’ | 155.14 |  | 154.3 |  |
| 4’ | 128.31 |  | 128.0 |  |
| 5’ | 154.27 |  | 153.7 |  |
| 6’ | 10.5 | 1.98, s | 10.5 | 2.00, s |
| 7’ | 59.99 | 3.63, s | 60.5 | 3.78, s |
| 8’ | 52.48 | 3.80, s | 53.0 | 3.85, s |
| **^a^** *Agric Biol Chem*. 1977, 41, 855-862 | | | | |

| **Table S3.** Comparison of the ^1^H and ^13^C data of **2** with the reported piericidin C1 | | | | |
| --- | --- | --- | --- | --- |
| **Position** | **2** (CD3CN) | | piericidin C1 **^a^** (CDCl_3_) | |
|  | *δ*_C_ (600 MHz) | *δ*_H_ (*J* in Hz) (150 MHz) | *δ*_C_ (100 MHz) | *δ*_H_ (*J* in Hz) (25MHz) |
| 1 | 35.0 | 3.34, d | 34.4 | 3.26, d |
| 2 | 123.1 | 5.37, t | 122.3 | 5.31, t |
| 3 | 136.0 |  | 134.9 |  |
| 4 | 43.5 | 2.78, d | 43.1 | 2.70, d |
| 5 | 126.8 | 5.60, m | 126.5 | 5.45, m |
| 6 | 137.1 | 6.06, d | 135.7 | 5.95, m |
| 7 | 134.9 |  | 134.6 |  |
| 8 | 135.1 | 5.24, d | 132.5 | 5.15, d |
| 9 | 37.4 | 2.61, t | 36.2 | 2.50, m |
| 10 | 82.6 | 2.81, d | 81.8 | 2.80, d |
| 11 | 63.1 |  | 62.6 |  |
| 12 | 58.5 | 2.86, q | 58.4 | 2.40, q |
| 13 | 13.6 | 1.24, d | 13.3 | 1.26, d |
| 14 | 11.4 | 1.19, s | 11.0 | 1.20, s |
| 15 | 17.6 | 0.87, d | 17.4 | 0.86, d |
| 16 | 16.8 | 1.73, s | 16.6 | 1.70, s |
| 17 | 13.2 | 1.74, s | 13.0 | 1.70, s |
| 1’ | 151.5 |  | 150.8 |  |
| 2’ | 113.3 |  | 112.2 |  |
| 3’ | 155.8 |  | 154.3 |  |
| 4’ | 129.2 |  | 127.9 |  |
| 5’ | 155.2 |  | 153.6 |  |
| 6’ | 10.8 | 2.04, s | 10.4 | 2.01, s |
| 7’ | 61.0 | 3.75, s | 60.5 | 3.78, s |
| 8’ | 53.4 | 3.88, s | 53.0 | 3.86, s |
| **^a^** *Agric Biol Chem*. 1977, 41, 855-862 | | | | |

| **Table S4.** Comparison of the ^1^H and ^13^C data of **3** with the literature one | | | | |
| --- | --- | --- | --- | --- |
| **Position** | **3** (DMSO-*d*_6_) | | Literature **^a^** (CD_3_OD) | |
|  | *δ*_C_ (600 MHz) | *δ*_H_ (*J* in Hz) (150 MHz) | *δ*_C_ (600 MHz) | *δ*_H_ (*J* in Hz) (150 MHz) |
| 1 | 173.0, C |  | 174.9, C |  |
| 2 | 33.3, CH_2_ | 2.96, d, (7.02) | 33.0, CH_2_ | 3.06, d, (7.2) |
| 3 | 117.3, CH | 5.30, m | 116.7, CH | 5.39, t, (6.6) |
| 4 | 137.8, C |  | 137.7, C |  |
| 5 | 42.2, CH_2_ | 2.75, d, (7.0) | 42.5, CH_2_ | 2.82, d, (7.2) |
| 6 | 124.0, CH | 5.46, dt, (15.5, 7.0) | 124.4, CH | 5.56, dt, (15.6, 7.2) |
| 7 | 136.5, CH | 6.03, d, (15.8) | 136.7, CH | 6.13, d, (15.6) |
| 8 | 132.1, C |  | 133.7, C |  |
| 9 | 135.4, CH | 5.28, m | 134.4, CH | 5.33, d, (9.6) |
| 10 | 36.1, CH | 2.54, dt, (9.4, 7.0) | 36.1, CH | 2.71, m |
| 11 | 80.5, CH | 3.61, m | 82.3, CH | 3.72, d, (7.8) |
| 12 | 136.7, C |  | 136.7, C |  |
| 12-OH |  | 4.47, d, (4.2) |  |  |
| 13 | 119.4, CH | 5.33, m | 121.4, CH | 5.47, q, (6.0) |
| 14 | 12.7, CH_3_ | 1.55, m | 11.7, CH_3_ | 1.64, d, (6.0) |
| 15 | 16.3, CH_3_ | 1.56 (3H, d, 1.3) | 15.0, CH_3_ | 1.66, s |
| 16 | 12.8, CH_3_ | 1.66 (3H, d, 1.2) | 11.7, CH_3_ | 1.78, m |
| 17 | 17.9, CH_3_ | 0.76, d, (6.8) | 16.7, CH_3_ | 0.83, d, (7.2) |
| 18 | 11.3, CH_3_ | 1.51, s | 9.7, CH_3_ | 1.63, s |
| **^a^** *Mar. Drugs* 2019, 17, 12; doi:10.3390/md17010012 | | | | |

| **Table S5.** ^1^H (500 MHz) and ^13^C (150 MHz) NMR data of **4** in DMSO-*d*_6_ | | |
| --- | --- | --- |
| **Position** | ***δ*_C_** | ***δ*_H_ (*J* in Hz)** |
| 1 | 173.1, C |  |
| 2 | 33.5, CH_2_ | 2.94, d, (7.1) |
| 3 | 117.6, CH | 5.28, m |
| 4 | 136.4, C |  |
| 5 | 42.2, CH_2_ | 2.74, d, (7.0) |
| 6 | 124.0, CH | 5.46, dt, (15.5, 7.0) |
| 7 | 136.5, CH | 6.03, d, (15.5) |
| 8 | 132.0, C |  |
| 9 | 135.3, CH | 5.28, m |
| 10 | 36.1, CH | 2.53, dt, (9.4,7.0) |
| 11 | 80.3, CH | 3.57, d, (7.1) |
| 12 | 134.4, CH |  |
| 13 | 133.0, CH | 5.08, dt, (9.1,1.2) |
| 14 | 26.2, CH | 2.47, m |
| 15 | 22.8, CH_3_ | 0.89, s |
| 16 | 16.2, CH_3_ | 1.56, s |
| 17 | 12.7, CH_3_ | 1.66, s |
| 18 | 17.9, CH_3_ | 0.77, d, (6.8) |
| 19 | 11.5, CH_3_ | 1.52, s |
| 20 | 23.0, CH_3_ | 0.90, s |

| **Table S6.** ^1^H (600 MHz) and ^13^C (150 MHz) spectroscopic data for compounds **5–7** | | | | | | |
| --- | --- | --- | --- | --- | --- | --- |
| **Position** | **5 ^b^** | | **6 ^a^** | | **7 ^a^** | |
|  | ***δ*_C_** | ***δ*_H_ (*J* in Hz)** | ***δ*_C_** | ***δ*_H_ (*J* in Hz)** | ***δ*_C_** | ***δ*_H_ (*J* in Hz)** |
| 1 | 29.50, CH_2_ | 3.15, d, (7.0) | 30.06, CH_2_ | 3.29, d, (7.0) | 30.07, CH_2_ | 3.28, d, (7.2) |
| 2 | 119.92, CH | 5.10, t, (7.0) | 118.49, CH | 5.20, m | 118.61, CH | 5.18, t, (7.2) |
| 3 | 135.96, C |  | 139.43, C |  | 139.04, C |  |
| 4 | 42.24, CH_2_ | 2.73, d, (7.0) | 43.07, CH_2_ | 2.80, d, (7.0) | 43.02, CH_2_ | 2.77, d, (7.0) |
| 5 | 124.13, CH | 5.43, m | 125.66, CH | 5.55, dt, (15.5, 7.0) | 125.62, CH | 5.53, dt, (15.5, 7.0) |
| 6 | 136.53, CH | 6.01, d, (15.5) | 136.58, CH | 6.09, d, (15.5) | 136.48, CH | 6.05, d, (15.5) |
| 7 | 132.08, C |  | 135.84, C |  | 135.03, C |  |
| 8 | 135.41, CH | 5.28, d, (9.8) | 133.84, CH | 5.23, d, (9.7) | 133.14, CH | 5.23, d, (9.5) |
| 9 | 36.09, CH | 2.52, m | 36.91, CH | 2.67, m | 36.36, CH | 2.62, m |
| 10 | 80.54, CH | 3.59, d, (7.3) | 82.94, CH | 3.62, d, (9.0) | 81.89, CH | 2.88, d, (8.9) |
| 11 | 137.77, C |  | 135.71, C |  | 62.62, C |  |
| 12 | 119.44, CH | 5.33, q, (6.7) | 123.63, CH | 5.47, m | 58.49, CH | 2.92, q, (5.5) |
| 13 | 12.82, CH_3_ | 1.54, d, (6.7) | 13.24, CH_3_ | 1.62, d, (6.7) | 13.39, CH_3_ | 1.28, d, (5.8) |
| 14 | 16.34, CH_3_ | 1.65, d, (7.6) | 16.70, CH_3_ | 1.70, s | 16.66, CH_3_ | 1.68 (3H, m) |
| 15 | 12.75, CH_3_ | 1.64, d, (7.6) | 13.28, CH_3_ | 1.78, dd, (5.8, 1.3) | 13.15, CH_3_ | 1.75 (3H, s) |
| 16 | 17.91, CH_3_ | 0.75, d, (6.8) | 17.50, CH_3_ | 0.80, d, (6.7) | 17.16, CH_3_ | 0.88, d, (6.8) |
| 17 | 11.31, CH_3_ | 1.50, s | 10.67, CH_3_ | 1.62, s | 11.02, CH_3_ | 1.27, s |
| 1' | 144.96, C |  | 143.54, C |  | 143.66, C |  |
| 2' | 103.89, C |  | 107.13, C |  | 106.96, C |  |
| 3' | 167.10, C |  | 169.40, C |  | 169.31, C |  |
| 4' | 96.08, CH | 5.45, s | 94.17, CH | 5.79, s | 94.16, CH | 5.77, s |
| 5' | 165.42, C |  | 165.34, C |  | 165.40, C |  |
| 6' | 9.25, CH_3_ | 1.78, s | 9.52, CH_3_ | 1.91, s | 9.47, CH_3_ | 1.89, s |
| 7' |  |  | 55.88, CH_3_ | 3.78, s | 55.83, CH_3_ | 3.78, s |
| **^a^** Recorded in CDCl_3_. **^b^** Recorded in DMSO-*d_6_*. | | | | | | |

**Figure S1.** HRMS analysis of compound **10** and predicted structure

**Figure S2.** SDS-PAGE analysis of recombinant PieB2

**
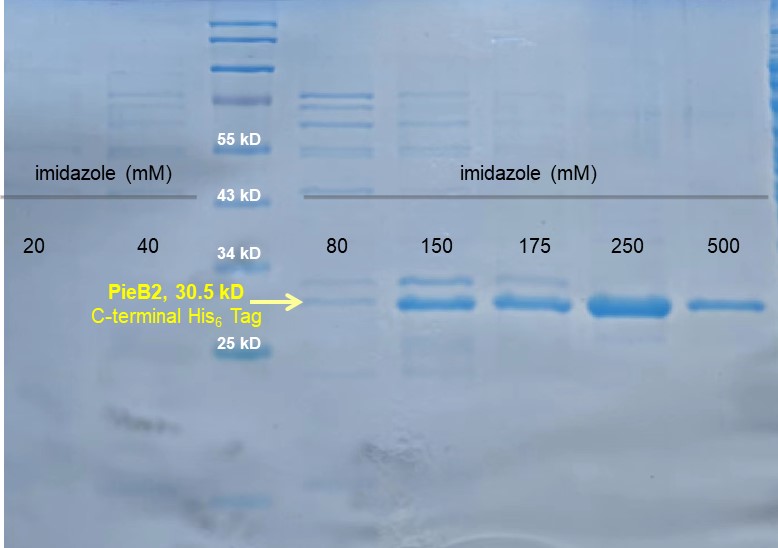
**

The target proteins were eluted by stepwise increases of the concentration of imidazole (10 to 500 mM)

**Figure S3.** Structural comparation of compounds **6** and piericidin N

**Figure S4.** NMR data for compound **1**

1. ^1^H NMR of compound **1** in DMSO-*d_6_* at 600 MHz

1. ^13^C NMR of compound **1** in DMSO-*d_6_* at 150 MHz

**Figure S5.** NMR data for compound **2**

1. ^1^H NMR of **2** in DMSO-*d_6_* at 500 MHz

1. ^13^C NMR of **2** in DMSO-*d_6_* at 125 MHz

**Figure S6.** Spectroscopic data for compound **3** (continued)

1. UV spectrum of compound **3**

**(B)** ^1^H NMR of compound **3** in DMSO-*d*_6_ at 600 MHz

**(C)** ^13^C NMR of compound **3** in DMSO-*d*_6_ at 125 MHz

**Figure S7.** Spectroscopic data for compound **4** (continued)

1. UV spectrum of compound **4**

**(B)** ^1^H NMR of compound **4** in DMSO-*d*_6_ at 500 MHz

**(C)** ^13^C NMR and DEPT 135 spectra of compound **4** in DMSO-*d*_6_ at 125 MHz

**(D)** HSQC spectrum of compound **4** in DMSO-*d*_6_ at 500 MHz

**(E)** ^1^H-^1^H COSY spectrum of compound **4** in DMSO-*d*_6_ at 500 MHz

**(F)** HMBC spectrum of compound **4** in DMSO-*d*_6_ at 500 MHz

**(G)** NOESY spectrum of compound **4** in DMSO-*d*_6_ at 500 MHz

**Figure S8.** Spectroscopic data for compound **5**

**(A)** UV spectrum of compound **5**

1. ^1^H NMR of compound **5** in DMSO-*d_6_* at 600 MHz

1. ^13^C NMR and DEPT 135 spectra of compound **5** in DMSO-*d_6_* at 150 MHz

**(E)** HSQC spectrum of compound **5** in DMSO-*d_6_* at 600 MHz

**(F)** ^1^H-^1^H COSY spectrum of compound **5** in DMSO-*d_6_* at 600 MHz

**(G)** HMBC spectrum of compound **5** in DMSO-*d_6_* at 600 MHz

**(H)** NOESY spectrum of compound **5** in DMSO-*d_6_* at 600 MHz

**Figure S9.** Spectroscopic data for compound **6** (continued)

1. UV spectrum of compound **6**

**(B)** ^1^H NMR of compound **6** in CDCl_3_ at 600 MHz

**(B)** ^13^C NMR and DEPT 135 spectra of compound **6** in CDCl_3_ at 150 MHz

**(C)** HSQC spectrum of compound **6** in CDCl_3_ at 600 MHz

**(D)** ^1^H-^1^H COSY spectrum of compound **6** in CDCl_3_ at 600 MHz

**(E)** HMBC spectrum of compound **6** in CDCl_3_ at 600 MHz

**(F)** NOESY spectrum of compound **6** in CDCl_3_ at 600 MHz

**Figure S10.** Spectroscopic data for compound **7** (continued)

1. UV spectrum of compound **7**

1. ^1^H NMR of compound **7** in CDCl_3_ at 600 MHz

**(C)** ^13^C NMR and DEPT 135 spectra of compound **7** in CDCl_3_ at 150 MHz

**(D)** HSQC spectrum of compound **7** in CDCl_3_ at 600 MHz

**(E)** ^1^H-^1^H COSY spectrum of compound **7** in CDCl_3_ at 600 MHz

**(F)** HMBC spectrum of compound **7** in CDCl_3_ at 600 MHz

**(G)** NOESY spectrum of compound **7** in CDCl_3_ at 600 MHz
